# Food insecurity and the dental team: a pilot study to explore opinions

**DOI:** 10.1038/s41405-024-00205-8

**Published:** 2024-03-18

**Authors:** Sondos Albadri, Lisa Allen, Teslimat Ajeigbe

**Affiliations:** 1 Professor and Honorary Consultant in Paediatric Dentistry, School of Dentistry, University of Liverpool, Liverpool, UK; 2 Academic Clinical Fellow NIHR Primary Dental Care, School of Dentistry University of Liverpool, Liverpool, UK; 3 Academic Clinical Fellow NIHR, School of Dentistry University of Liverpool, Liverpool, UK

**Keywords:** Nutrition and diet in dentistry, Health care

## Abstract

**Purpose:**

In total, 17% of UK households with children experience food insecurity, with evidence to suggest a direct correlation with the prevalence of oral disease. This study explores current perceptions of the dental team, when recognising and supporting families who may experience food insecurity.

**Materials and methods:**

An online, anonymous cross-sectional survey was designed and sent to members of the British Society of Paediatric Dentistry (BSPD) in June 2023, examining confidence and understanding surrounding food insecurity and dental health. Quantitative data is presented descriptively and qualitative data using a thematic analysis.

**Results:**

The response rate was 9.6% (*n* = 76). A significant number recognise the link between poor oral health and food insecurity, 80.3% (*n* = 61). Although practitioners are confident in oral health counselling, 80.3% (*n* = 61) a smaller proportion are not as confident when approaching food insecurity 32.9% (*n* = 25). Dental team members recognise the need to improve identification of affected patients and that they have a professional duty to support. Intervention strategies, such as additional training to support team development and signposting of patients are indicated.

**Conclusion:**

This study suggests that whilst dental professionals understand the link between food insecurity and oral health, and their responsibilities to those affected; they lack confidence in identifying such patients and providing support. Additional conversations and training are fundamental to better understand their role, which must reflect the needs of the population that they serve.

## Introduction

The oral health survey of 2022 [1] detailed that the national prevalence of enamel or dentinal decay in 5-year-old children was 29.3% with wide regional variation from 23.3% in the Southwest to 38.7% in the Northwest. Dental disease, despite being a preventable disease, continues to be the largest contributor to hospital admissions throughout the UK in children; and in 2021 to 2022 there were 26,741 episodes of tooth extractions in NHS hospitals for 0–19year olds with a primary diagnosis of dental caries, costing the NHS £50.9 million [2].

Food insecurity is defined as a lack of the financial resources needed to ensure reliable access to food to meet dietary, nutritional, and social needs [3]. It includes families or individuals who struggle to fill their shopping bags, pay for food, and prepare a nutritious meal for everyone living in their home adequately. In June 2023, it was estimated that currently 3.7 million children are said to live in a food insecure household [4]. Across all households in the UK, food and non-alcoholic drink is the fourth most significant household expenditure after housing, transport, and recreation and culture [5]. Thus demonstrating, that with rising cost of energy and housing prices, this is continuing to decrease household food budgets. It is estimated currently that those in the poorest fifth of UK households would need to spend 50% of their current disposable income to meet the current government UK health diet needs [6]. Overall, food organisations agree that current levels of income are insufficient to sustain a healthy and equitable diet as advised by government policy and consequently, food insecurity is considered an independent predictor of dental caries in both adolescents and children [7].

In recent years, several systematic reviews have been conducted which indicate that there is a direct correlation between food insecurity and dental disease [7–9]. Families who experience food insecurity may choose cheaper, energy dense foods to supplement their diet which have reduced nutritional value and are higher in fat and sugar [8]. As a result, studies would suggest that children in a situation of food insecurity may have more untreated dental caries [10–12], and dental pain resulting from caries [13] as well as greater prevalence rates of restorations and extractions [14]. Furthermore, severe enamel hypoplasia and chronic periodontal disease are associated with a lack of vitamin D [15], and scorbutic gingivitis (scurvy) is associated with vitamin C deficiency [16]. Such diseases influence the cost of future provisions within dental services and the wider NHS.

While there has been some investigatory work to consider the views of medical professionals regarding food insecurity [17], there is little information regarding the confidence of dental professionals in this area. Evidence suggests that even when discussing oral health and aiming for co-designed treatment plans, dental professionals still lead by offering patients a range of options that they consider to be in the patients’ best interests and what the patient ‘needs’ [18] rather than tailoring advice to individual circumstances. It is recognised how important it is to appreciate the varied factors which influence patients’ behaviours, which will help dental professionals adopt realistic expectations of patients’ scope for change. [19]. This is especially true for those experiencing food insecurity.

The aim of this study was to explore current awareness and understanding of food insecurity within the dental team. It examines the levels of confidence and understanding amongst the team regarding the importance of general dental health education. More specifically, it explores current levels of awareness and confidence in the provision of care for those who experience food insecurity and considers perceived barriers, as well as options for further support.

## Materials and methods

A cross-sectional study using an anonymous, voluntary, self-administered online questionnaire (Supplementary File 1) was designed and ethical approval was granted by the University of Liverpool ethics committee (Ethical approval number 12323).

Prior to commencement of the study, a search of the literature was conducted, and no validated questionnaire was found. An online questionnaire was designed consisting of 21 questions. Prior to dissemination, the questionnaire was peer reviewed and piloted by local dental staff, and feedback was used to inform questionnaire development.

A combination of closed-ended and open-ended questions were employed to gather both quantitative and qualitative data, and to facilitate coding and data interpretation. Closed-ended questions included mixed-item response formats, which were exhaustive and mutually exclusive. Five-point Likert scales (A-Strongly disagree, B- Disagree, C-Neutral, D-Agree, E-Strongly agree) were incorporated to determine participant confidence and understanding in relation to current provision of dental health education and more specifically in relation to food insecurity.

The questionnaire and covering letter were sent to the lead administrator of the BSPD via email who forwarded this on the study teams’ behalf, throughout the month of June 2023 to 698 members of the BSPD. A second email reminder was sent after two weeks of initial contact.

Inclusion criteria involved health professionals currently undertaking clinical sessions with children and members of the BSPD. Those practitioners not regularly treating children, or administerial staff not currently undertaking a clinical role were excluded from the survey response as it was felt that they would not have direct experience with our target population. It was understood that most members would meet the inclusion criteria which was outlined within the information leaflet sent to members, and in this situation all respondents satisfied the requirements. This was verified on analysis of survey data.

Quantitative data were exported into Microsoft Excel 2019 for descriptive statistics and presented graphically for visual display with analysis of trends. Qualitative data were exported into NVivo 12 software for thematic analysis. Data was coded by the lead researcher and initial themes were generated. Themes were discussed and further refined by a second researcher with qualitative research experience and agreed by the research team.

## Results

In total seventy-six dental professionals completed the survey online, a response rate of 9.6%. Findings have been collated and presented in response to our research objectives.

### Demographics

Respondents ranged in age between 18 and 64, the majority being in the 25–34 age range (51.3% *n* = 39) with an even representation from 35 to 64 years of age. 90.8% were female (*n* = 69) and 9.2% male (*n* = 7). Many respondents (35.5% *n* = 27) had qualified 6–10 years previously.

### Primary role and primary place of work

The largest proportion of respondents worked within the Hospital dental service 55.3% (*n* = 42) and Community dental services 40.8% (*n* = 31) with a small proportion of respondents working within general dental practise. 3.9% (*n* = 3).

Professional roles varied widely. Consultants and speciality registrars within the Hospital dental services make up the greatest proportion of responders 44.7% (*n* = 34), followed by representatives from Community dental services 35.4%, (*n* = 27).

Respondents were predominantly dentists 96% (*n* = 73) with 2.6% response from dental therapists (*n* = 2) and one dental student.

### Provision of Oral health education

Most respondents felt that diet is a major component in oral health 94.7% (*n* = 72), whilst 5.3% disagreed (*n* = 4). Similarly, 96% (*n* = 73) of respondents felt that it is the responsibility of dental professionals to provide dietary counselling, with 80.3% (*n* = 61) of respondents reporting that they have adequate knowledge to provide this advice. There were a small number who were less confident in provision of oral health education (19.7%, *n* = 15).

80.3% of respondents (*n* = 61), were aware of the impact of food insecurity on oral health yet only 36.8% of respondents (*n* = 28) had the confidence to identify individuals that may be experiencing food insecurity and 32.9% (*n* = 25) were comfortable discussing this further.

### Responsibilities as dental professionals

Many of the respondents 81.6% (*n* = 62) agreed that dental teams have a role in advising patients who experience food insecurity. Four respondents disagreed with this statement.

When asked to leave free comments, some respondents indicated that there are more appropriate services to aid patients:

“I don’t think this is my job to have these discussions. There are other more appropriate services who can provide this information.”

“I do not believe it is the role of dentists to act as social workers. I would of course direct families I felt were struggling to services which may help with this, but I do not feel that dentists should be the ones to be leading these discussions.”

### Barriers

**Fig. 1:**
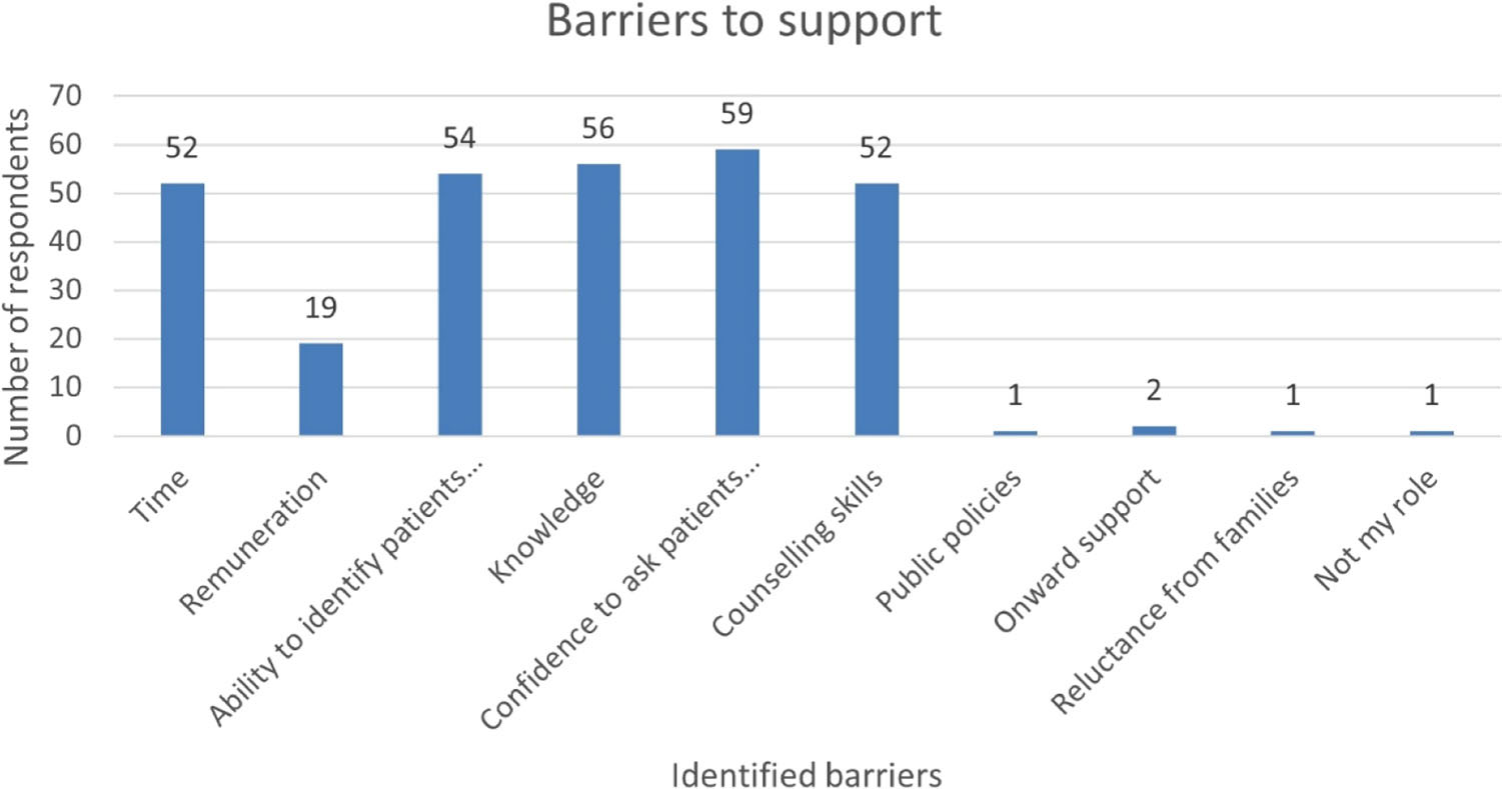
Frequency of respondents indicating which barriers they encounter when discussing Food Insecurity. Answers to free text responses.

Figure 1 demonstrates that of the perceived barriers when discussing food insecurity with families. This included lack of time 65.8% (*n* = 50), lack of confidence to ask patients 77.6% (*n* = 59), and difficulties in identification of patients 71% (*n* = 54). Lack of knowledge 73.4% (*n* = 56) and counselling skills 68.4% (*n* = 52) was also identified as a barrier.

Other factors identified were remuneration, lack of public policies, onward support, reluctance from families to discuss and not within my role.

### Support for the dental team

In total, 32.9% (*n* = 25) of respondents were aware of services that families with food insecurity can access and 18.4% (*n* = 14) were able to signpost for further support.

### Thematic analysis

**Fig. 2:**
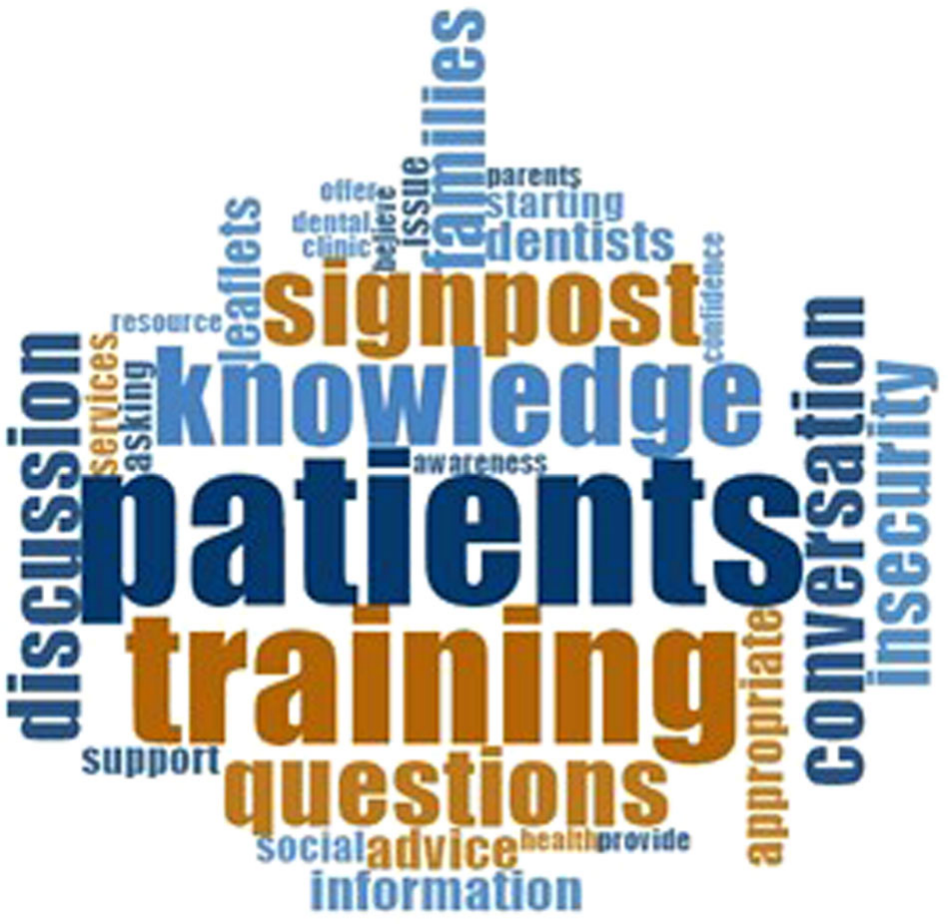
Word map. Word map to demonstrate common themes in qualitative responses.

The word map (Fig. 2) demonstrates the main themes emerging when asked, what would enable the dental team to initiate a conversation regarding food insecurity?

#### Patients

Respondents identified the need to communicate appropriately and sensitively with suggestions such as:

“Just be open and ask as part of the assessment” and “If parents and children are engaged and relaxed, they may well talk about food insecurity. Being sensitive to the fact that people with food insecurity may feel stigmatised whilst appreciating the “rights of the child” “and “being able to offer practical, realistic and holistic advice is very important in a non-judgemental way.”

There was the need to discuss this topic in a structured way to screen appropriately and ensure consistency in approach, for example “Agreeing a set question to ask that wouldn’t offend anyone.” And “I think you need a sentence that you can use directly as a question to all patients as part of your history taking, that you use regularly so that you feel comfortable saying it, much like asking about social care involvement or raising the issues of very high or very low BMI.”

One respondent highlighted the need to tailor diet advice commenting “I do worry about the lack of knowledge and skills around cooking and nutrition are adding to problems in everyday life. I think nutrition and basic cooking skills should be required for all children in secondary education. However, if a family is unable to afford to cook from scratch it is important to be sensitive. I get very concerned when colleagues talk about sugar in baked beans as an oral health problem when it is irrelevant as such foods are highly nutritious cheap and ok to eat cold if the electricity has been cut off.”

#### Training

There was a recognition amongst respondents that additional training in this area is needed before they are confident to discuss further, ranging from “training by an expert in the field about how to tackle a difficult conversation i.e., how to frame the question and to know what to ask / give appropriate advice” with a need for training in counselling and role play.

Additional training within the Undergraduate and Postgraduate training curriculum was also suggested by one respondent. “I always bring up socioeconomic issues with trainees and ways to ask questions respectfully in order to help patients/parents. Many trainees are from affluent backgrounds and have literally no idea how many of our patients live. I have over the years developed ways to ask kindly and without judgement if patients have toothbrushes or have a poor diet and trainees/undergrads should have this as part of their training”.

#### Resources

Respondents suggested the need for posters in waiting rooms, information leaflets and understanding of onward referrals with one suggesting, “Having a good resource that I can then signpost the patient to. It feels quite empty if we start a conversation and give advice, but then cannot signpost to the appropriate services. If I had a website / leaflet / pathway that I can then signpost the patient to I would have more confidence in starting the discussion.”

Again, a small number of respondents felt this was not within their role and one respondent highlighted the need for “More media and government coverage - social acceptance and knowledge on food insecurity”.

### Differences based on time since graduation

**Fig. 3:**
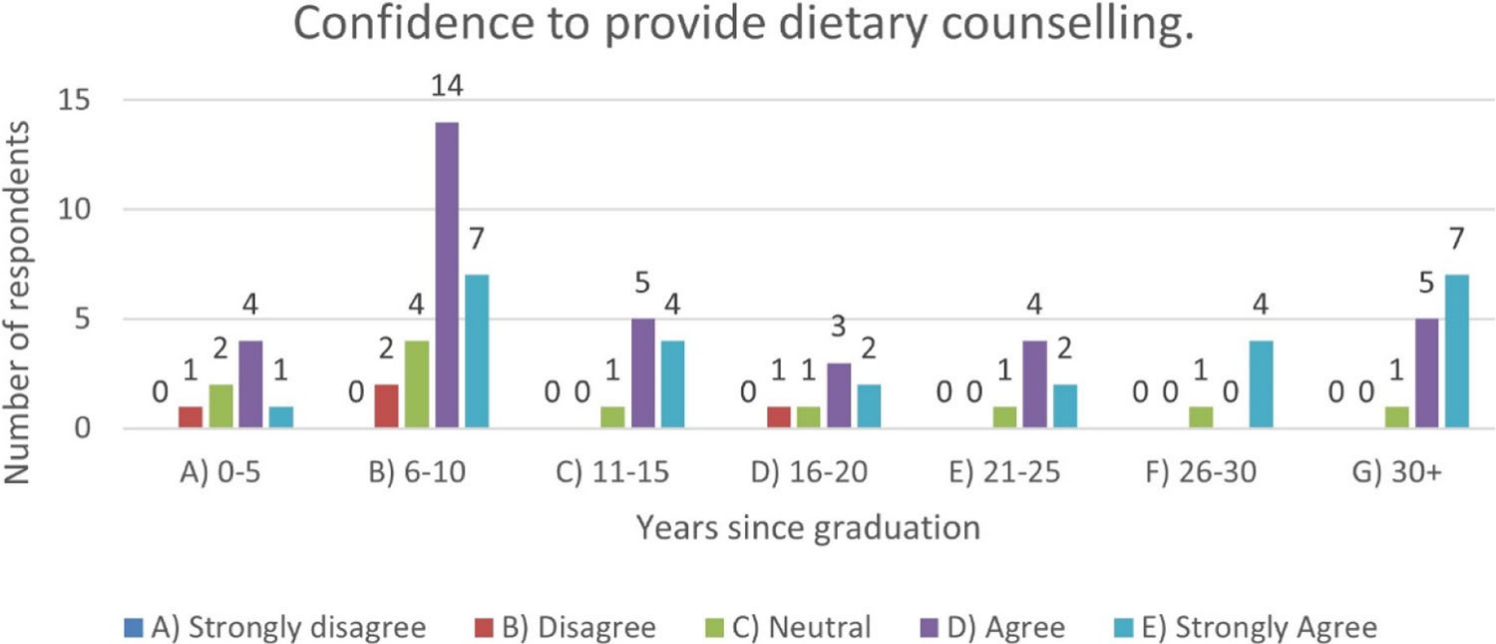
Number of respondents having the confidence to provide dietary counselling vs years since graduation. Answers to Likert Scale responses.

Finally, differences were explored based on number of years’ experience and confidence levels. Respondents across all ranges were confident in providing dietary counselling. (Fig. 3).

Figure 3 demonstrates that a larger proportion of respondents are confident to provide dietary counselling across each category, however more respondents disagreed with the statement who had recently qualified in the last 10 years 5.3% (*n* = 4). This is a small number however and cannot be used to generalise across the group. All groups believe that dental health education is important with a small minority early in their career 0-10 years 5.3% (*n* = 3) who do not believe that this is the case.

**Fig. 4:**
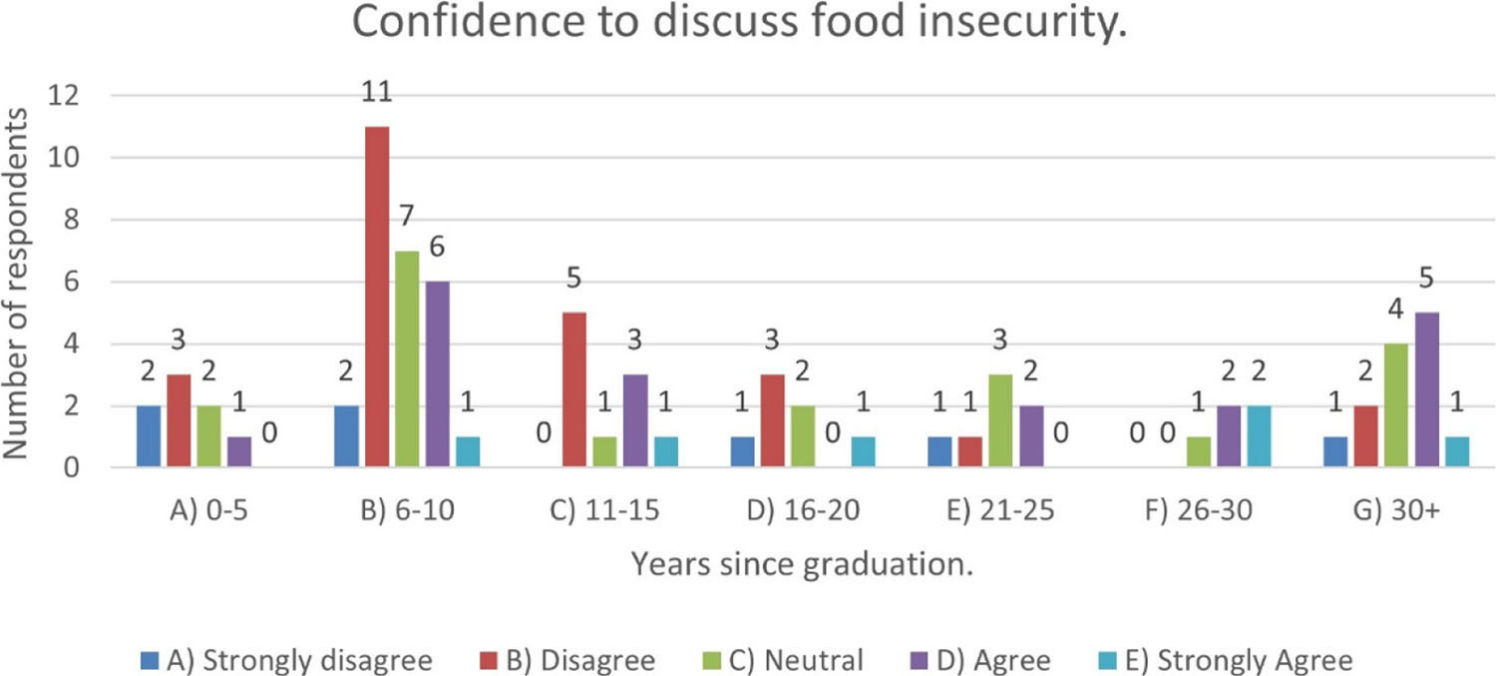
Number of respondents who were confident to discuss food insecurity within families vs years qualified. Answers to Likert Scale responses.

Figure 4 demonstrates, there is a greater proportion of the group who would disagree or strongly disagree that they have the confidence to discuss food insecurity, and this is consistent across all age ranges.

Again, there appears to be a varied opinion as to levels of responsibility especially in the cohort recently qualified.

## Discussion

It is believed that 14.4 million of the UK population live in poverty including 4.2million children [20]. Food prices have increased drastically during the cost-of-living crisis while wages and benefits have not kept up. This has led to levels of food insecurity doubling over the course of 2022 [21]. Hungry children struggle to achieve, and poor nutrition can damage their general health leading to obesity, malnutrition, and other non-communicable diseases such as dental caries. Whilst there have been other studies to address support given by other healthcare professionals [22] in relation to food insecurity, we were unable to identify any such research conducted within the dental profession.

Within the UK, children are eligible for free NHS dental treatment and are encouraged to attend their dentist to detect dental disease at an early stage and promote confidence within the dental environment. It is currently estimated that there are 7 million child dental appointments yearly [23]; which gives the dental profession an immense opportunity to identify families who may be struggling with finances and unknown to other services when they may for example attend complaining about cost of transport to access their appointment or with pain as a result of dental decay.

Our study shows there is a need for dental professionals to better understand those experiencing food insecurity and how to support their care. A team approach is advocated to ensure that all those directly working with dental patients have a better understanding and can support appropriately in a sensitive and professional manner.

The target group identified were BSPD members as they were more likely to have some experience treating children from areas of deprivation, and therefore at increased risk of Food Insecurity. Subsequently, findings from this study raise several significant points for consideration.

### Confidence of the dental team

It is evident that many respondents were confident in their approach to providing general oral health education to the public and that they had the knowledge and experience to do so. Dental health professionals are taught to follow best practise to improve the oral health of our patients. “Delivering better oral health” highlights that the UK population is consistently eating too many “free sugars” which should not exceed greater than 5% of total dietary energy from 2 years upwards [24]. It refers practitioners to resources such as the Eatwell guide and Change4Life which have clear guidance about eating a healthy diet. [25, 26].

When we consider the cost of fruit and vegetables, these are the most expensive “Eatwell Guide” food category by a significant margin, costing on average £10.56 per 1000 kcal. In comparison, foods high in fat, sugar and/or salt (HFSS) are considerably cheaper, costing on average just £4.50 per 1000 kcal [27]. Therefore, it is likely that people living within food insecure households base their food choices on quantity rather than quality which would lead to an increased uptake of refined carbohydrates, fats, and sugars [28] and cheaper alternatives such as processed foods. This can lead to a significant impact on both physical and mental health and lead to not only dental disease but malnutrition, obesity, heart disease and diabetes as a result [28].

One third of the study respondents were confident that they were able to identify individuals that may experience food insecurity. This may be largely due to this being a sensitive and stigmatising topic and the reluctance to shame or embarrass patients by asking the question directly. Proposals such as screening for all patients via links or medical history screening could therefore be potentially explored.

Additionally, some dentists felt that they had a trusting relationship with patients to discuss further, whilst showing empathy and compassion. Proposals such as coaching skills could be explored to empower clinicians to initiate this conversation.

### Professional responsibility

While many respondents felt that the dental team had a responsibility to support their patients, our results highlighted that a minority of dentists felt that this was not part of their role. One of the respondents raised a key point as to how they encourage all trainees to understand social and economic determinants of health as this underpins people’s lives and health conditions [29].

The UK government and NHS encourage all healthcare providers to work collaboratively to improve health and encourage healthy behaviour changes to reduce the risk of future disease. One such example is the making every contact count initiative [30] which aims to reduce health inequalities, by maximising every contact with health professionals for health benefit. The subject of food insecurity should align with smoking and alcohol cessation advice as an example in the same approach.

Interestingly, those stating that they did not feel this was their role had qualified within the past 20 years. It may be of interest to explore if this is a product of current education or that understanding evolves with life experience.

### Proposed intervention strategies

Finally, how can the profession support those in need? Cost of living is at an all-time high with food prices rising significantly. Whilst there is an understanding of the need to focus on a good diet for oral and general health, beyond this, it is difficult to understand what interventions are most effective.

There were many positive practical suggestions to aid patients who may be experiencing food insecurity including screening, provision of resources and signposting into the community. All require further consideration and consultation directly with patients to prevent feeling of shame or stigmatisation. A collaborative approach with those patients who have lived experiences of food insecurity is needed to consider their priorities and codesign interventions.

### Support for health professionals

It is apparent that currently little information is provided to the dental profession surrounding food insecurity and oral health, as well as the need to advocate change on a national level. Further research should be conducted in this area which would support educational development for the future, including identification of current curriculum topics within the undergraduate curriculum and areas for development.

### Study limitations and strengths

Unfortunately, as with many questionnaires, the response rate was too small to generalise results across the entire target group. Despite this, our qualitative data provides a deeper understanding from those who are likely to work with this subgroup.

Respondents were predominantly dentists and therefore views of the wider dental care professionals were limited. However, results retrieved were consistent with membership demographics.

To give a 95% confidence interval and enable generalisation of results, a response rate of 235 was desirable. Therefore, this study should be viewed as a pilot study. The low response rate may be due to a short window of opening for the survey or that members did not feel the subject a priority. Also, the month of distribution was June when many members may be taking their summer holidays. If this area is revisited, these factors should be taken into account, considering the duration of survey opening as well as the time of year. As those respondents had an interest in child dentistry and work largely within the NHS it is important however to highlight the need for further support in this area and explore further.

## Conclusion

The study is the first to investigate the views of dental professionals in this area.

Without disregarding the present already overstretched workforce and the need for a change in public health policies, it is important to develop reliable professional practise within the dental team and support our most vulnerable patients. This should be in conjunction with those directly affected and ensure ethical practise is followed. The need to understand and offer support to those experiencing food insecurity has never been more critical and further research should be conducted to explore the views of patients and dentists in more detail.

### Supplementary information

Supplementary Information

## Data Availability

Data is available upon request.
