# Supplementary material for: Food insecurity and the dental team: a pilot study to explore opinions
Source: BDJ Open. 2024 Mar 18;10:24. doi: 10.1038/s41405-024-00205-8 (PMC10948798; doi:10.1038/s41405-024-00205-8)
Supplement: Supplementary file 1 — Supplementary Information [file 41405_2024_205_MOESM1_ESM.pdf]

Supplementary File 1: Survey to BSPD Members

Consent to participate (To be sent within Microsoft forms link)

Participant consent form

Version number & date:

Research ethics approval number:

Title of the research project:

Name of researcher(s):

Please tick box.

1. I confirm that I have read and have understood the information sheet dated [**DATE**] for the above study, or it has been read to me. I have had the opportunity to consider the information, ask questions and have had these answered satisfactorily. ☐

2. I understand that taking part in the study involves a short online questionnaire. ☐

3. I understand that my participation is voluntary and that I am free to stop taking part and can withdraw from the study at any time without giving any reason and without my rights being affected. In addition, I understand that I am free to decline to answer any particular question or questions. ☐

4. I understand that the information I provide will be held securely and in line with data protection requirements at the University of Liverpool until it is processed and then deposited in the archive for sharing and use by other authorised researchers to support other research in the future. ☐

5. I understand that signed consent forms and original **questionnaires** will be kept securely for up to ten years on the password protected hard drive of the principle investigator and then will be destroyed. ☐

6. I agree to take part in the above study. ☐

Please tell us about yourself.

1: What is your age group?

A) 18—24
B) 25-34
C) 35-44
D) 45-54
E) 55-64
F) 65 or older

2: Number of years since graduation?

A) 0-5
B) 6-10
C) 11-15
D) 16-20
E) 21-25
F) 26-30
G) 30+

3: Which of the following best describes your gender?

A) Male
B) Female
C) Non-Binary
D) Prefer not to say.

4: **Which of the following best describes your primary clinical role?**

A) Hospital dental services -a: Consultant
b: Speciality and Associated specialist.
c: Speciality registrar
d: Clinical Teaching role
e: Dental core trainee
f: Clinical Assistant

B) Community dental services a: Medical director/consultant
b: Band C clinical specialist
c: Band B clinician
d: Band A clinician
e: Dental Therapist
f: Other

C) General dental practise a: Principal
b: Associate with specialist interest paediatric dentistry
c: Associate dentist
d: Foundation dentist
e: Therapist
f: Hygienist
g: Foundation therapist
h: Other Allied Health professional

Current Diet advice provided:

How much would you agree with the following statements, strongly disagree/Disagree/Neutral/Agree/Strongly agree.

5: Diet is a major component in oral health

A) Strongly Disagree
B) Disagree
C) Neutral
D) Agree
E) Strongly Agree

6: It is the responsibility of dental professions to provide dietary counselling.

A) Strongly disagree.
B) Disagree
C) Neutral
D) Agree
E) Strongly Agree

7: I have the knowledge to provide dietary counselling

A) Strongly disagree.
B) Disagree
C) Neutral
D) Agree
E) Strongly Agree

8: I have the confidence to provide dietary counselling

A) Strongly disagree.
B) Disagree
C) Neutral
D) Agree
E) Strongly Agree

In relation to food insecurity:

9: I am aware of the impact of food insecurity on oral health

A) Strongly disagree.
B) Disagree
C) Neutral
D) Agree
E) Strongly Agree

10: I have the confidence to identify individuals experiencing food insecurity.

A) Strongly disagree.
B) Disagree
C) Neutral
D) Agree
E) Strongly Agree

11: I am confident to discuss food insecurity and oral health within families

A) Strongly disagree.
B) Disagree
C) Neutral
D) Agree
E) Strongly Agree

12: I am aware of services that families with food insecurity can access.

A) Strongly disagree.
B) Disagree
C) Neutral
D) Agree
E) Strongly Agree

13: I am able to signpost families to services available that support families with food insecurity.

A) Strongly disagree.
B) Disagree
C) Neutral
D) Agree
E) Strongly Agree

14: Dental teams have a role in advising patients who experience food insecurity.

A) Strongly disagree.
B) Disagree
C) Neutral
D) Agree
E) Strongly Agree

Barriers

15: What do you think may be potential barriers may be in relation to managing patients with food insecurity? (Please tick)

Time
Repatriation
Ability to identify patients who would benefit.
Knowledge
Confidence to ask patients about food insecurity.
Counselling skills
Other Please list---

16: What do you think could help you initiate a conversation around food insecurity?

17: Please add any additional comments you feel are relevant

Thank you for taking the time to submit your responses.
